# Lack of Association of the Polymorphisms *IL-17A* (−197G/A) and *IL-17F* (+7488A/G) with Multibacillary Leprosy in Mexican Patients

**DOI:** 10.1155/2014/920491

**Published:** 2014-11-06

**Authors:** Mónica Escamilla-Tilch, Iris Estrada-García, Julio Granados, Roberto Arenas-Guzmán, Rosalio Ramos-Payan, Thalía Gabriela Pérez-Suárez, Ma. Isabel Salazar, Riky Luis Pérez-Lucas, Sergio Estrada-Parra, Nora Magdalena Torres-Carrillo

**Affiliations:** ^1^Departamento de Inmunología, Escuela Nacional de Ciencias Biológicas, Instituto Politécnico Nacional, 11340 México, DF, Mexico; ^2^Instituto Nacional de Geriatría, Periférico Sur No. 2767, Col. San Jerónimo Lídice, Del. Magdalena Contreras, 10200 México, DF, Mexico; ^3^Departamento de Trasplantes, División de Inmunogenética, Instituto Nacional de Ciencias Médicas y Nutrición Salvador Zubirán, 14000 México, DF, Mexico; ^4^Sección de Micología, Hospital General Dr. Manuel Gea González, 14080 México, DF, Mexico; ^5^Facultad de Ciencias Químico Biológicas, Universidad Autónoma de Sinaloa, 80010 Culiacán, SIN, Mexico

## Abstract

*Background.* Leprosy is a chronic infectious disease caused by the intracellular acid-fast bacilli *Mycobacterium leprae*; it has been determined that genetic factors of the host play an important role in the disease susceptibility. Thus, in this case-control study, we evaluated the possible association between the *IL-17A G-197A* (rs227593) and *IL-17F A7488G* (His161Arg, rs763780) gene SNPs and susceptibility to leprosy disease in Mexican population. *Methods.* Seventy-five leprosy patients and sixty-nine control subjects were included. Both SNPs were genotyped with the polymerase chain reaction-restriction fragment length polymorphism technique. *Results.* We found nonsignificant differences in genotype and allele frequencies related to *IL-17A G-197A* (rs227593) and *IL-17F A7488G* (His161Arg, rs763780) gene SNPs in MB as well as subclinical forms of leprosy disease versus healthy individuals. *Conclusions.* Since the sample size is not large enough, it is difficult to sustain an association of susceptibility to leprosy with genotypes or allele frequencies of *IL-17A G-197A* (rs227593) and *IL-17F A7488G* (His161Arg, rs763780), suggesting that *IL-17* polymorphisms have no significant role in the genetic susceptibility to development of this disease in the Mexican Mestizo population.

## 1. Introduction

Leprosy is a human chronic infectious disease caused by the intracellular acid-fast bacilli* Mycobacterium leprae*. It affects the skin and peripheral nerves of susceptible individuals, causing irreversible impairment of nerve function and consequent chronic disability [1]. Despite the global coverage of multidrug therapy promoted by the World Health Organization, 215 new cases of leprosy were reported in Mexico until 2012, with a prevalence of 0.480 cases per every 100,000 inhabitants. In addition, the proportion of multibacillary (MB) cases has increased in some Mexican population associated with a declining incidence [2, 3].

The clinical spectrum of leprosy includes two poles, the lepromatous (LL) and tuberculoid (TT), with several intermediate (I) or dimorphic forms, including borderline tuberculoid (BT), mid-borderline (BB), and borderline lepromatous (BL); furthermore, in the WHO classification standards, the BB, BL, and LL individuals belong to multibacillary (MB) patients, whereas the BT and TT individuals go to paucibacillary (PB) patients. Immunologically, the LL skin lesions are distinguished by predominance of CD8^+^ T cells and it is characterized by a Th2 T-cell immune response, with an antibody complex formation that is unable to contain the infection. By contrast, the TT skin lesions are characterized by a predominance of CD4^+^ T cells; they present a Th1 T-cell cytokine reaction with a vigorous T-cell response that restricts the* M. leprae* growth [4–6].

On the other hand, this balance between the Th1 (TT) and Th2 (LL) response can be influenced by a third participant, the Th17 cells, which are known to produce a proinflammatory cytokine called interleukin-17 (IL-17) [7]. IL-17 is a newly described cytokine that bonds the adaptive and innate immune systems. So far, six IL-17 family ligands (IL-17A-F) and five receptors (IL-17RA-RD and SEF) have been identified. IL-17A and IL-17F are the members of the IL-17 cytokine family responsible for the pathogenic activity of the Th17 cells; this cytokines family induce multiple proinflammatory mediators, including chemokines, cytokines, and metalloproteinases, from epithelial and fibroblast cells [8].

Based on the biological relevance of IL-17A and IL-17F cytokines, in this study we analyzed two single-nucleotide polymorphisms (SNPs) within the* IL-17A* and* IL-17F* genes including the G-197A (rs227593) and A7488G (His161Arg, rs763780), respectively, which have been identified to be associated with several diseases [9]. Therefore, taking into consideration that the genetic background of patients influences the immune response to acquire the infection and consequently provides different clinical manifestations [10], the aim of this study was to analyze the association between the* IL-17A* and* IL-17F* genes SNPs with susceptibility or resistance to develop leprosy in Mexican patients.

## 2. Materials and Methods 

### 2.1. Subjects

In this study, we recruited a total of 75 patients with leprosy (41% female and 59% male, mean age of 53.9 ± 18.7 years) which were classified according to the International Criteria established by Ridley and Jopling [4]. The patients were residents from the states of Sinaloa (33.3%), San Luis Potosí (25.3%), Puebla (17.4%), Distrito Federal (9.3%), Guanajuato (8%), and Yucatán (6.7%). Sixty-two patients were classified as LL, 9 as dimorphic (D), 2 as TT, and 2 as I. All leprosy cases were MB, with exception of 4 PB. As a control group, we included 69 healthy subjects (55.5% female and 44.5% male, mean age of 32.2 ± 12.8 years), unrelated to the patients and matched by ethnicity. Ethnically, patients and controls were classified as Mestizos, who are defined as those individuals born in Mexico that have a Spanish last name, with Mexican ancestors at least back to the third generation. Mestizos are the result of 500 years of admixture between Spaniards, Amerindians, and Africans, and they currently represent most of the Mexican population (>90%) [11, 12]. The informed written consent was obtained from all subjects before enrollment to the study, according to the ethical guidelines of 2008 Declaration of Helsinki.

### 2.2. Genotyping

For genotyping, venous blood samples of leprosy patients and controls were collected into EDTA-containing tubes, and the genomic DNA (gDNA) was extracted from peripheral blood leukocytes according to Miller's salting-out method [13]. The* IL-17A* G-197A (rs227593) and* IL-17F* A7488G (His161Arg, rs763780) gene polymorphisms were analyzed by the polymerase chain reaction-restriction fragment length polymorphism system (PCR-RFLP) which was slightly modified from the two previously described methods [14]. The PCR amplification was performed in a total volume of 30 *μ*L, containing 100 ng of gDNA, 1 *μ*M of each oligonucleotide, 0.2 mM of each dNTPs, 2 mM of MgCl_2_, 0.5 U/*μ*L of Taq DNA polymerase, and supplied buffer enzyme 1X (Invitrogen Life Technologies, Carlsbad, CA, USA). The amplification conditions were 95°C for 3 min, 35 cycles of 95°C for 30 s, 56°C for 30 s, 72°C for 30 s, and a final extension of 72°C for 3 min for* IL-17A *G-197A and for* IL-17F* A7488G they were 95°C for 3 min, 35 cycles of 95°C for 30 s, 60°C for 30 s, 72°C for 30 s, and a final extension of 72°C for 3 min. The PCR products were digested at 37°C for 2 h with 1 U of* XagI* and* NIaIII *restriction enzymes (New England BioLabs, Beverly, MA, USA) for the identification of the* IL-17A* G-197A (rs227593) and* IL-17F* A7488G (His161Arg, rs763780) gene polymorphisms, respectively. Finally, the digested PCR products were electrophoresed on 3% agarose gels ethidium bromide stained for the identification of the genotypes both polymorphisms.

### 2.3. Statistical Analysis

Frequencies of genotypes and alleles, Hardy-Weinberg Equilibrium (HWE), and OR with a 95% CI were estimated using the Chi-square test (*χ* ^2^) (Epi Info statistical software 3.3.2, Atlanta Georgia). Comparison data were evaluated by *χ* ^2^ test or Fisher's exact test when applicable. The* P* values were adjusted with Bonferroni correction for multiple testing (*P*_*c*_).

## 3. Results

We investigated the possible association with leprosy in 144 Mexican Mestizo individuals (75 leprosy patients and 69 healthy controls) who were genotyped for the* IL-17A* G-197A (rs227593) and* IL-17F* A7488G (His161Arg, rs763780) gene polymorphisms. For this purpose and taking into account that the majority of cases of leprosy in Mexico are MB [3, 15], we only considered the MB leprosy and control group for this analysis. The distribution of genotypic and allelic frequencies of* IL-17A* G-197A (rs227593) and* IL-17F* A7488G (His161Arg, rs763780) gene polymorphisms in patients with leprosy and healthy controls is shown in Table 1. Both SNPs were in HWE in the control group (*P* > 0.05; data not shown). The distribution of genotypic and allelic frequencies between MB leprosy patients and control group did not show significant differences in* IL-17A* G-197A (rs227593) and* IL-17F* A7488G (His161Arg, rs763780) gene polymorphisms (*P* > 0.05). This lack of association was confirmed when we performed the analysis in the clinical subtypes (LL and D) of MB leprosy, where we did not find significant differences (*P* > 0.05; Table 2).

## 4. Discussion

Despite global coverage of multidrug therapy promoted by the World Health Organization the leprosy remains as a public health problem [2]. Nowadays, we know that certain individuals who are exposed to the* M. leprae* develop leprosy, suggesting that genetic background is relevant in the development of it, since it determines the control of the immune response against this infection [16]. In this study we aimed to identify the genotypic and allelic frequencies of* IL-17A* G-197A (rs227593) and* IL-17F* A7488G (His161Arg, rs763780) gene SNPs in the context of leprosy to identify if there was any association with susceptibility to leprosy in the Mexican Mestizo population. We did not find an association between both* IL-17A* G-197A (rs227593) and* IL-17F* A7488G (His161Arg, rs763780) gene SNPs and MB leprosy or their clinical subtypes, so our study provides evidence of lack of association with susceptibility in leprosy patients; moreover, the genotype and alleles frequencies have not been reported previously in Mexican population.

This observation differs with a study in the North India Cohort, where the* IL-17F* A7488G (His161Arg, rs763780) gene SNP is associated with susceptibility to leprosy; likewise, they found that AG genotypes confer a decreased risk of acquiring leprosy in the North Indian Cohort and observed that this SNP also influences the clinical phenotypes in leprosy disease [17], and to our knowledge, this is the first study that reported association of this polymorphism with leprosy susceptibility. To date, there are some studies related to* IL-17A* G-197A (rs227593) and* IL-17F* A7488G (His161Arg, rs763780) gene SNPs and risk of tuberculosis (TB), gastric cancer, rheumatoid arthritis (RA), intestinal bowel disease (IBD), Crohn's disease (CD), ulcerative colitis (UC), and others [14]. In this previous studies, patients from a Chinese Han population who had the genotype AG/AA of* IL-17F* A7488G (His161Arg, rs763780) gene SNP were more susceptible to acquiring tuberculosis, compared to the GG genotype [18], whereas in other studies of the China population it was verified that the* IL-17F* A7488G (His161Arg, rs763780) gene SNP was associated with risk of gastric cancer and GA genotype was associated with the clinic-pathological features and not with the risk of gastric cancer itself. However, in the case of the* IL-17A* G-197A (rs227593) gene SNP, this polymorphism was not associated with the risk of gastric cancer [14]. Moreover, in Iranian population it was observed that the* IL-17A* G-197A (rs227593) gene SNP was significantly associated with the risk of gastric cancer, since patients who had the homozygous AA were 2.9 times more likely to develop disease; furthermore, the presence of a single A allele increases the risk of gastric cancer up to 1.7-fold. This association was only observed in early stage gastric adenocarcinomas and was not linked to* H. pylori* infection [19]. On the other hand, the close association of* IL-17A* G-197A (rs227593) gene SNP with susceptibility to the development of osteoarthritis (OA) in the Korea population has been described; however, they did not find relationship between the* IL-17F* A7488G (His161Arg, rs763780) gene SNP and OA susceptibility [20]. With respect to IBD which includes CD and UC, it was reported in German population that the* IL-17F* A7488G (His161Arg, rs763780) gene SNP is not considered as a marker of susceptibility for IBD per se but is associated with increased disease activity [21], whereas in Chinese population it was demonstrated that the* IL-17F* A7488G (His161Arg, rs763780) gene SNP has no effect on susceptibility to DC but is associated with protection to UC [22]. The discrepancy in the association of* IL-17A* G-197A (rs227593) and* IL-17F* A7488G (His161Arg, rs763780) gene SNPs could be due to the type of pathology studied but mainly to the frequency at which the polymorphic allele in each population is presented, which reflects the genetic heterogeneity of the different population, suggesting that the low frequency or absence of the polymorphic alleles may confer an advantage of decreasing risk of susceptibility. Moreover, environmental and socioeconomic factors may influence the development of different pathologies. In conclusion, we identify lack of association of* IL-17A* G-197A (rs227593) and* IL-17F* A7488G (His161Arg, rs763780) gene SNPs with susceptibility to leprosy in Mexican population.

## Figures and Tables

**Table 1:** Allelic and gene frequencies of polymorphisms in *IL-17A* and *IL-17F* genes in patients with multibacillary leprosy and healthy controls.

	MB patients	CS	OR (95% CI)	*P* value
	*n* (%)	*n* (%)		
Genotype				

*IL-17A *−197	*N* = 68	*N* = 69		

GG	38 (56)	29 (42)	—	—
GA	30 (44)	40 (58)	0.57 (0.27–1.19)	2.11
AA	0 (0)	0 (0)		
GA + AA	30 (44)	40 (58)	0.57 (0.27–1.19)	2.11

*IL-17F *+7488	*N* = 71	*N* = 68		

AA	58 (82)	52 (76)	—	—
AG	13 (18)	16 (24)	0.73 (0.30–1.7)	0.58
GG	0 (0)	0 (0)		
AG + GG	13 (18)	16 (24)	0.73 (0.30–1.7)	0.58

Allele				

*IL-17A *−197	*N* = 136	*N* = 138		

G	106 (78)	98 (71)	—	—
A	30 (22)	40 (29)	0.96 (0.39–1.24)	0.24

*IL-17F *+7488	*N* = 142	*N* = 136		

A	129 (91)	120 (88)	—	—
*G *	13 (9)	16 (12)	0.76 (0.33–1.74)	0.60

The values are presented as frequency in percentage (%) and number (*n*) of genotypes or alleles. The frequencies comparison between groups was analyzed by Chi-square test. Statistical significance was at *P* < 0.05 Yates corrected. OR, odds ratio; CI, confidence interval; *N*, number of individuals; MB, multibacillary leprosy; CS, healthy controls.

**Table 2:** Allelic and gene frequencies of polymorphisms in *IL-17A* and *IL-17F* genes in subclinical forms (LL and D) and healthy controls.

	D patients *n* (%)	CS *n* (%)	OR (95% CI)	^ a^ *P* value	LL patients *n* (%)	OR (95% CI)	^ b^ *P* value	OR (95% CI)	^ c^ *P* value
Genotype									

*IL-17A *−197	*N* = 8	*N* = 69			*N* = 60				

GG	7 (88)	29 (42)	—	—	31 (52)	—	—	—	—
GA	1 (12)	40 (58)	0.10 (0–0.93)	0.038	29 (48)	1.47 (0.69–3.14)	0.358	0.15 (0.01–1.38)	0.123
AA	0 (0)	0 (0)			0 (0)				
GA + AA	1 (12)	40 (58)	0.10 (0–0.93)	0.038	29 (48)	1.47 (0.69–3.14)	0.358	0.15 (0.01–1.38)	0.123

*IL-17F *+7488	*N* = 9	*N* = 68			*N* = 62				

AA	7 (78)	52 (76)	—	—	51 (82)	—	—	—	—
AG	2 (22)	16 (24)	1.08 (0.17–8.38)	0.739	11 (18)	1.43 (0.56–3.67)	0.551	1.32 (0.16–8.64)	0.891
GG	0 (0)	0 (0)			0 (0)				
AG + GG	2 (22)	16 (24)	1.08 (0.17–8.38)		11 (18)	1.43 (0.56–3.67)	0.551	1.32 (0.16–8.64)	0.891

Allele									

*IL-17A *−197	*N* = 16	*N* = 138			*N* = 120				

G	15 (94)	98 (71)	6.12 (0.80–1228)	0.039	91 (76)	0.21 (0.01–1.63)	0.192	1.28 (0.71–2.32)	0.464
A	1 (6)	40 (29)			29 (24)				

*IL-17F *+7488	*N* = 18	*N* = 136			*N* = 124				

A	16 (89)	120 (88)	1.07 (0.20–7.39)	0.757	113 (91)	1.28 (0.0–7.08)	0.897	1.37 (0.57–3.32)	0.575
*G *	2 (11)	16 (12)			11 (9)				

The values are presented as frequency in percentage (%) and number (*n*) of genotypes or alleles. The frequencies comparison between groups was analyzed by Chi-square test. Statistical significance was at *P* < 0.05 Yates corrected; OR, odds ratio; CI, confidence interval; *N*, number of individuals; MB, multibacillary leprosy; CS, healthy controls; D, dimorphic; LL, lepromatous leprosy. ^a^D patients versus CS; ^b^LL versus CS; ^c^D versus LL.
